# Anti-Inflammatory Effect of *Chamaecyparis obtusa* (Siebold & Zucc.) Endl. Leaf Essential Oil

**DOI:** 10.3390/molecules29051117

**Published:** 2024-03-01

**Authors:** Sung-Hee Kim, Young-Ah Jang, Yong-Jin Kwon

**Affiliations:** 1 Department of Cosmeceutical Science, Kyungsung University, Busan 48434, Republic of Korea; 2 Division of Cosmetic Science, Daegu Haany University, Gyeongsan 38610, Gyeongbuk, Republic of Korea; 3 Department of Cosmetic Science, Kyungsung University, Busan 48434, Republic of Korea

**Keywords:** *Chamaecyparis obtusa* (Siebold &amp Zucc.) Endl. (*C. obtusa*), sabinene, macrophage, anti-inflammatory

## Abstract

*Chamaecyparis obtusa* (Siebold & Zucc.) Endl. (*C. obtusa*) belongs to the Cupressaceae family and is native to East Asian regions. Essential oils extracted from the leaves, bark, branches, and roots of *C. obtusa* have both aesthetic and medicinal properties and are thus widely used. However, detailed analyses of the active ingredients of *C. obtusa* extract are lacking. In this study, the sabinene content in the hydro-distillation of *C. obtusa* leaf essential oil (COD) was analyzed using GC-MS, and the anti-inflammatory effect of COD was compared with that of pure sabinene. Cell viability was evaluated by MTT assay, and nitric oxide (NO) production was measured using Griess reagent. Relative mRNA and protein levels were analyzed using RT-qPCR and western blot, and secreted cytokines were analyzed using a cytokine array kit. The results showed that both COD and sabinene inhibited the expression of inducible nitric oxide synthase (iNOS) and the phosphorylation of c-Jun N-terminal kinase (JNK) and p38 in lipopolysaccharide (LPS)-induced RAW 264.7 cells. COD and sabinene also reduced the production of pro-inflammatory cytokines interleukin (IL)-1β, IL-6, IL-27, IL-1 receptor antagonist (IL-1ra), and granulocyte-macrophage colony-stimulating factor (GM-CSF). The anti-inflammatory mechanisms of COD and sabinene partially overlap, as COD was shown to inhibit MAPKs and the JAK/STAT axis, and sabinene inhibited MAPKs, thereby preventing LPS-induced macrophage activation.

## 1. Introduction

Inflammation is a complex immunological defense mechanism that protects organisms from pathogenic infections or injuries [1]. It is an essential immune response that maintains tissue homeostasis under various adverse conditions [2]. Acute inflammation is self-regulated through mechanisms involving the downregulation of pro-inflammatory proteins and upregulation of anti-inflammatory proteins [3]. However, chronic inflammation can lead to cardiovascular diseases, rheumatoid arthritis, autoimmune disorders, neurological conditions, respiratory diseases, and cancer [4].

Macrophages are immune cells that also play a role in the inflammatory response by phagocytizing pathogens. Toll-like receptor 4 (TLR4), expressed by macrophages, is activated by lipopolysaccharide (LPS) originating from the cell wall of gram-negative bacteria. Upon activation, TLR4 triggers downstream signaling pathways, including nuclear factor (NF)-κB and mitogen-activated protein kinases (MAPKs) [5]. This activation leads to the translocation of transcription factors into the nucleus and, thus, the synthesis and subsequent release of pro-inflammatory cytokines, such as interleukin (IL)-1β, IL-6, and tumor necrosis factor (TNF)-α [6]. These molecules activate the Janus kinase/signal transducer and activator of transcription (JAK/STAT) signaling pathway and increase the expression of the pro-inflammatory enzyme inducible nitric oxide synthase (iNOS), leading to excessive nitric oxide (NO) production [7].

Among the pharmacological inhibitors targeting inflammation and cancer are inhibitors of MAPKs (e.g., SP600125, PD98059, SB203580) and JAK/STAT signaling processes (e.g., AG-490, nifuroxazide, S3I-201) [8,9,10,11]. However, as these inhibitors often induce clinically relevant side effects, there is growing interest in the use of natural compounds with lower toxicity and fewer side effects. For example, depending on the solvent used for extraction, several natural compounds exhibit activity attributed to secondary metabolites or their derivatives, such as alkaloids, flavonoids, phenols, allelochemicals, quinones, and volatile aromatic compounds [12]. Essential oils, which are highly fragrant, volatile natural compounds, are used in perfumes, cosmetics, and natural medicines. Because their active components are able to penetrate the skin, essential oils have been used to remove skin impurities, prevent skin aging, and promote skin regeneration [13].

**Figure 1:**
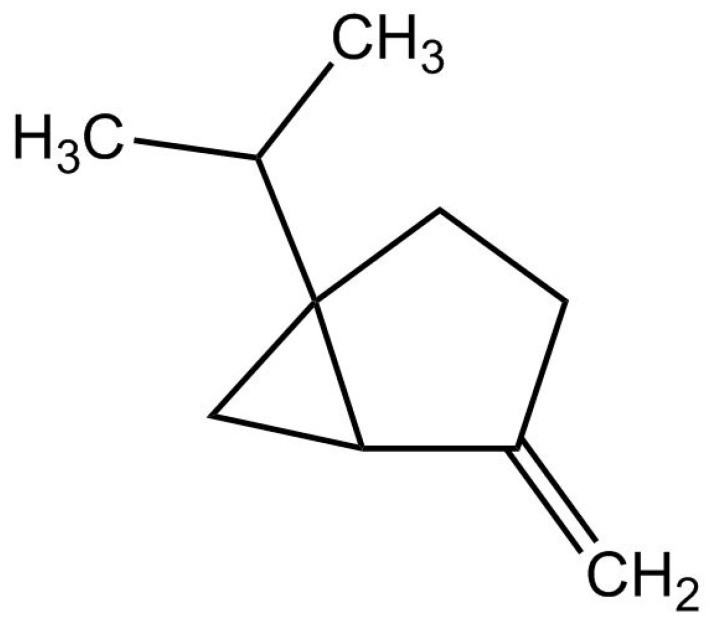
Chemical structure of sabinene.

*Chamaecyparis obtusa* (*C. obtusa*) belongs to the Cupressaceae family and is native to East Asia, including Korea, China, and Japan. Due to its high-quality wood and unique fragrance, *C. obtusa* has found many applications, and the essential oils obtained from its leaves, bark, branches, and roots are common ingredients in air fresheners and bath products. The essential oil extracted from *C. obtusa* leaves contains phytoncides, which have been shown to enhance psychological stability and improve cardiovascular and pulmonary functions [14]. Previous studies have investigated the anti-inflammatory mechanisms of *C. obtusa* depending on the extraction method and plant part, the skin-brightening effect of *C. obtusa* bark extract, and the promotion of hair growth in animal models [15,16,17]. However, the active ingredients of *C. obtusa* extracts have not been directly compared. In a previous study, gas chromatography-mass spectrometry (GC-MS) analysis of *Daucus carota* L. ssp. *carota* essential oil showed that the sabinene content was (28.2–37.5%), the highest among the active substances, and was used as the main active substance in the study [18]. Sabinene was reported as the main compound in essential oils extracted from various parts of *Juniperus sabina* and *Juniperus foetidissima*, and it was found to have the highest content in essential oils from all parts of *Juniperus sabina* [19]. In addition, the sabinene content of *Oenanthe crocata* L. essential oil was 29%, which was confirmed as a major anti-inflammatory substance, but its immune activity was not further investigated [20]. Sabinene is a scented monoterpene compound with a ring structure and is mainly found in herbs, trees, and the essential oils of plants (Figure 1; PubChem CID: 18818) [21]. Therefore, we focused on sabinene, which has a high content in most essential oils, and conducted a comparative analysis with *C. obtusa* leaf essential oil. In this study, the sabinene content of *C. obtusa* leaf essential oil obtained by hydro-distillation (COD) was analyzed, and the anti-inflammatory mechanism of COD was compared with that of pure sabinene.

## 2. Results

### 2.1. GC-MS Analysis of COD

GC-MS analysis of COD showed that monoterpenes (65.3%) and sesquiterpenes (31.5%) were present in large amounts. The main monoterpenes were β-myrcene (3.6%), limonene (6.9%), γ-terpinene (3.9%), terpinen-4-ol (2.7%), bornyl acetate (10.7%), and α-terpinyl acetate (18.7%) (Table 1). A substantial amount of sabinene was detected (12.3%). Thus, in subsequent experiments, we compared the activity of COD with that of sabinene.

### 2.2. Cell Viability of COD- and Sabinene-Pretreated RAW 264.7 Cells

**Figure 2:**
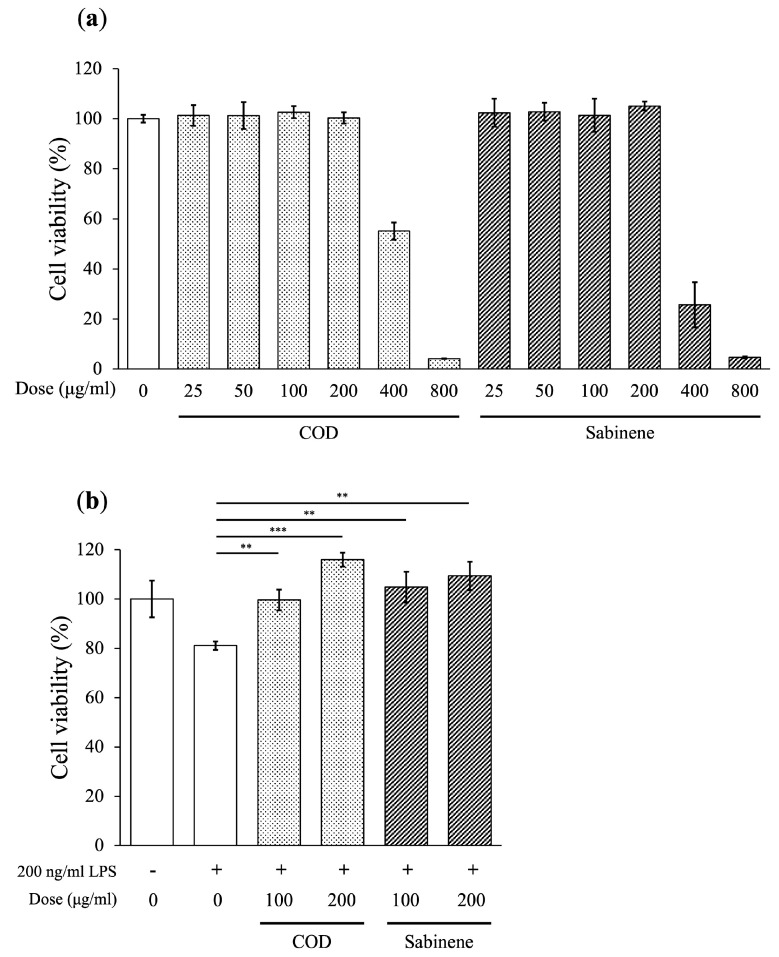
Cell viability of COD- and sabinene-pretreated RAW 264.7 cells. The cytotoxicity and cell protective ability of COD and sabinene were evaluated in a cell viability (MTT) assay. (**a**) Cytotoxicity of COD and sabinene in RAW 264.7 cells. The cells were treated for 24 h with COD or sabinene at concentrations of 25, 50, 100, 200, 400, and 800 μg/mL. (**b**) COD and sabinene increase the viability of LPS-induced RAW 264.7 cells. LPS-induced RAW 264.7 cells were treated with COD and sabinene at concentrations of 100 and 200 μg/mL for 2 h, followed by 200 ng/mL lipopolysaccharide (LPS) for 16 h. Data represent the mean ± SD, *n* = 3. ** *p* < 0.01 and *** *p* < 0.001 vs. LPS-treated group by Student’s *t*-test.

The cytotoxicity and cell protective ability of COD and sabinene were evaluated in a cell viability (MTT) assay using RAW 264.7 cells and LPS-induced RAW 264.7 cells, respectively. In the cytotoxicity assay, cells were treated for 24 h with COD or sabinene at concentrations of 25, 50, 100, 200, 400, and 800 μg/mL. The results showed a cell viability of >90% at concentrations ≤200 μg/mL (Figure 2a). LPS-induced RAW 264.7 cells were treated with COD and sabinene at concentrations of 100 and 200 μg/mL for 2 h followed by 200 ng/mL LPS for 16 h. Cell viability was 81.1% in the LPS-only group, whereas in cells pretreated with COD and sabinene, a dose-dependent increase in cell viability ranging from 99.62% to 115.97% and 104.85% to 109.87%, respectively, was observed (Figure 2b). These results suggest that COD and sabinene exhibit anti-inflammatory effects as they prevent LPS-induced cell death.

### 2.3. Anti-Inflammatory Properties of COD and Sabinene in LPS-Induced RAW 264.7 Cells

**Figure 3:**
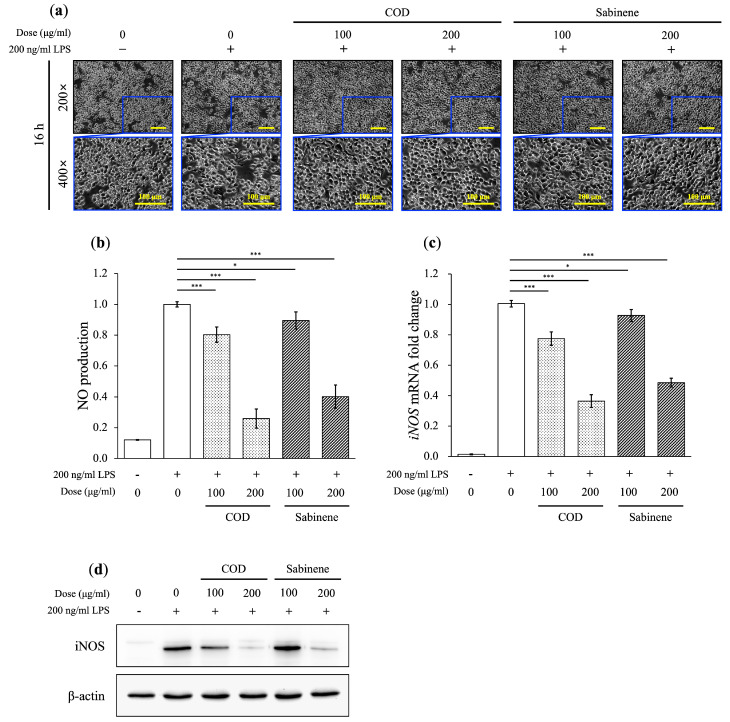
Anti-inflammatory properties of COD and sabinene in LPS-induced RAW 264.7 cells. Cells were pretreated for 2 h with COD or sabinene at concentrations of 100 and 200 μg/mL, followed by 200 ng/mL LPS for 16 h. (**a**) Representative images of the cells are shown. Scale bars: 100 μm. (**b**) NO levels were determined using Griess reagent. (**c**,**d**) Relative mRNA expression was analyzed by RT-qPCR (**c**) and relative protein expression by western blot (**d**). All expression data were calculated relative to *β-actin* expression. Data represent the mean ± SD, *n* = 3. * *p* < 0.05 and *** *p* < 0.001 vs. LPS-treated group by Student’s *t*-test.

The morphological changes in LPS-induced RAW 264.7 macrophages were compared with those described in previous studies, which confirmed cellular activation. These changes were dose-dependently alleviated by both COD and sabinene, as observed by microscopy (Figure 3a). A dose-dependent reduction in NO production by LPS-induced RAW 264.7 cells pretreated with COD and sabinene was also determined (Figure 3b). The latter finding was supported by the comparable dose-dependent decrease in the levels of iNOS mRNA and protein expression in pretreated cells (Figure 3c,d). These results highlight the similar anti-inflammatory effects of COD and sabinene.

### 2.4. Reductions in Pro-Inflammatory Cytokine Levels by COD and Sabinene in LPS-Induced RAW 264.7 Cells

**Figure 4:**
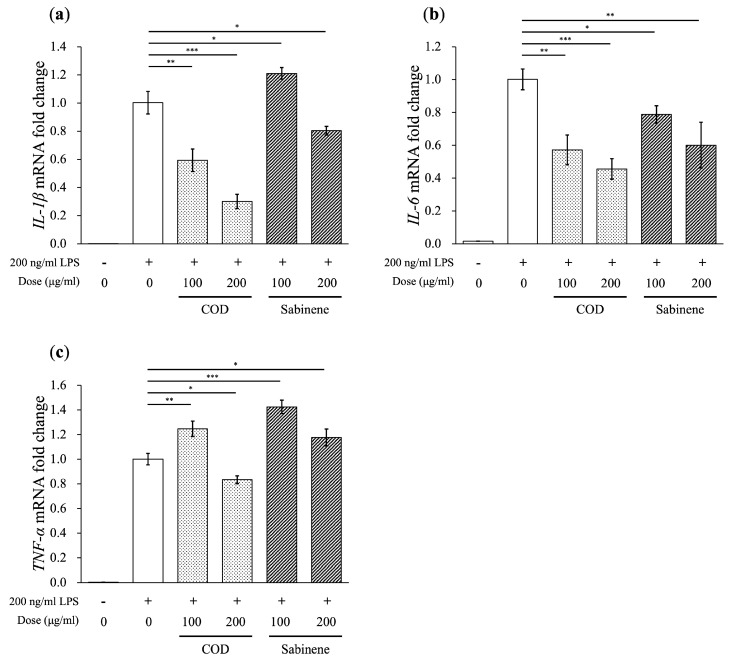
Reduction in pro-inflammatory cytokine levels by COD and sabinene in LPS-induced RAW 264.7 cells. (**a**–**c**) Cells were pretreated for 2 h with COD or sabinene at concentrations of 100 and 200 μg/mL, followed by 200 ng/mL LPS for 4 h. Relative mRNA expression was analyzed using RT-qPCR. All expression data were calculated relative to *β-actin* expression. Data represent the mean ± SD, *n* = 3. * *p* < 0.05, ** *p* < 0.01, and *** *p* < 0.001 vs. LPS-treated group by Student’s *t*-test.

The mechanism underlying the anti-inflammatory effects of COD and sabinene was explored by measuring the expression of the pro-inflammatory cytokines *IL-1β*, *IL-6*, and *TNF-α*, activated by LPS stimulation, in pretreated RAW 264.7 cells [22]. COD significantly reduced the mRNA levels of *IL-1β* and *IL-6*, whereas sabinene reduced the mRNA levels of *IL-6* by more than half but had no effect on *IL-1β*, even at high concentrations (Figure 4a,b). Neither COD nor sabinene significantly inhibited the expression of *TNF-α* (Figure 4c).

### 2.5. Evaluation of MAPKs, NF-κB/IκBα, and the JAK/STAT Axis in COD- and Sabinene-Pretreated LPS-Induced RAW 264.7 Cells

**Figure 5:**
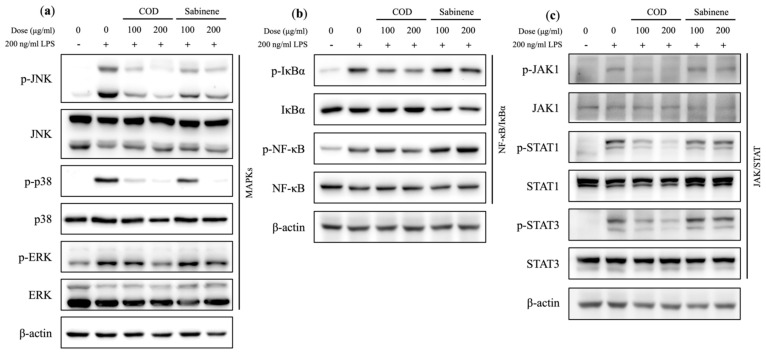
Evaluation of MAPKs, NF-κB/IκBα, and the JAK/STAT axis in COD- and sabinene-pretreated LPS-induced RAW 264.7 cells. (**a**–**c**) Cells were pretreated for 2 h with COD or sabinene at concentrations of 100 and 200 μg/mL, followed by 200 ng/mL LPS for 4 h. Relative protein expression was analyzed by western blot.

The anti-inflammatory mechanisms of COD and sabinene were further investigated by examining representative downstream signaling molecules that induce the expression of pro-inflammatory cytokines. Both COD and sabinene significantly reduced the activation of phosphorylated JNK and p38 in LPS-induced RAW 264.7 cells, whereas ERK activation was reduced only by COD (Figure 5a). COD also reduced the activation of phosphorylated IκBα, JAK 1, and STAT 1 and 3 (Figure 5b,c). In contrast, sabinene had no effect on the phosphorylation of ERK, NF-κB/IκBα, or JAK/STAT (Figure 5a–c). These results suggest that COD inhibits the inflammatory response of LPS-induced macrophages through MAPKs and the JAK/STAT axis, while sabinene inhibits the inflammatory response of LPS-induced macrophages through MAPKs.

### 2.6. Cytokine Array Analysis in COD- and Sabinene-Pretreated LPS-Induced RAW 264.7 Cells

**Figure 6:**
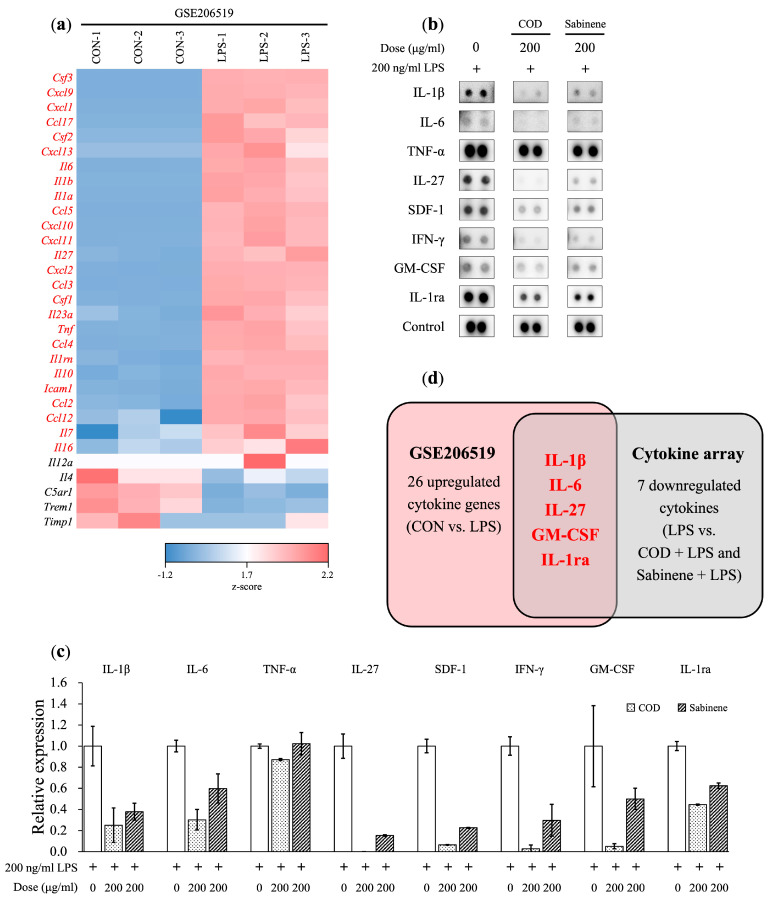
Cytokine array analysis in COD- and sabinene-pretreated LPS-induced RAW 264.7 cells. (**a**) Heatmap of mRNA expression of genes in control (CON) and LPS-treated (LPS) RAW 264.7 cells, based on the GSE206519 database. Z-scores were calculated for each gene and visualized; blue represents low expression, and red represents high expression. (**b**,**c**) Cells were pretreated for 2 h with COD or sabinene at a concentration of 200 μg/mL, followed by 200 ng/mL LPS for 4 h. (**b**) Expressed cytokines were analyzed using a cytokine array and (**c**) cytokine blots were quantified using ImageJ bundled with Java 8. (**d**) Illustration showing 26 upregulated cytokine genes (CON vs. LPS) according to GSE206519 and seven downregulated cytokine genes (LPS vs. COD + LPS and sabinene + LPS) in the cytokine array. Five downregulated cytokines were those whose expression decreased in response to both COD and sabinene.

The expression of pro-inflammatory cytokines in inflammatory diseases is well established [23]. Given the demonstrated expression of pro-inflammatory *IL-1β*, *IL-6*, and *TNF-α* in LPS-induced RAW 264.7 cells at the mRNA level and the results of our analysis of downstream signaling molecules, a cytokine array was performed. Based on the GSE206519 database set [24], 26 cytokines were upregulated, and 7 cytokines were downregulated in LPS-induced RAW 264.7 cells (Figure 6a–c and Figure S1). Both COD and sabinene downregulated five cytokines: IL-1β, IL-6, IL-27, granulocyte–macrophage colony-stimulating factor (GM-CSF), and IL-1 receptor antagonist (IL-1ra) (Figure 6d). IL-27 promotes NO production in LPS-induced macrophages and activates MAPKs, NF-κB, and JAK/STAT signaling [25]. Phagocytes activated by GM-CSF cause tissue damage [26]. Although IL-1ra is an anti-inflammatory cytokine, it plays a major role in acutely amplifying pancreatic or systemic inflammation, and it has been reported that the level of IL-1ra increases as the severity of acute pancreatitis increases [27]. As a result of the cytokine array analysis showed that COD and sabinene significantly reduced the expression of the pro-inflammatory cytokines IL-1β and IL-6, as well as IL-27, GM-CSF, and IL-1ra.

## 3. Discussion

The inflammatory response is an important physiological mechanism that protects the human body from pathogens and toxic external stimuli [1]. However, the adverse effects of prolonged expression of inflammatory genes and mediators that occur in chronic inflammation have been implicated in many diseases [3]. Signaling pathways and molecules activated by the immune system and the inflammatory response, such as NF-κB, MAPKs, and JAK/STAT, mediate cell proliferation, differentiation, apoptosis, and other cellular processes, in addition to regulating the expression of genes activated by cellular stresses such as LPS [5,7].

NF-κB exists in several homodimer and heterodimer forms, including NF-κB1 (p50/p105), NF-κB2 (p52/p100), RelA (p65), c-Rel, and RelB. While p65 acts as a transcriptional activator, p50 and p52 are transcriptional repressors [28]. Binding to inhibitor κBα (IκBα) inhibits the activity of the NF-κB dimer, such that it is present in an inactivated state in the cytoplasm [29]. The IκB kinase (IKK) complex degrades IκBα through phosphorylation and polyubiquitination, thus releasing NF-κB from IκBα and causing its activation [30]. When NF-κB is phosphorylated, it enters the nucleus, where it activates or suppresses the expression of specific genes, thus regulating various cellular functions [28]. MAPKs are composed of independent signaling molecules, including JNK, ERK, and p38, and their dysregulation can lead to uncontrolled cell growth and proliferation, hence their association with many diseases, including cancer [31,32]. The phosphorylation of MAP3K by Ras-GTP initiates a protein kinase cascade [33]. Activated MAPKs enter the nucleus together with NF-κB and phosphorylate various transcription factors, resulting in the release of pro-inflammatory cytokines that activate JAK/STAT molecules, which also mediate gene transcription [1]. The JAK/STAT pathway participates in immune responses and inflammation-related gene expression [7]. IL-1β expression is reduced by inhibiting the activation of MAPKs, whereas IL-6 binds to cell surface receptors to promote the activation of STAT3. Activation of the NF-κB pathway increases TNF-α gene expression [34,35,36]. Given this intertwined relationship between pro-inflammatory cytokines and downstream signaling molecules, inhibitors designed to inhibit the expression of any one of them have multiple effects and are typically highly toxic. This has led to a greater interest in natural products, with their potential for fewer side effects. In a previous study, the anti-inflammatory effect of *C. obtusa* essential oil was investigated through the cyclooxygenase-2 pathway in rats [37]. In addition, sabinene has been reported to be the main compound of various essential oils such as *Citrus reticulate* Blanco and *Zornia diphylla* (L.) Pers, and in a previous in vivo study, sabinene was reported that injection of 1% sabinene suppressed lens protein-induced inflammation in rabbit eyes [38,39]. Therefore, in this study, we investigated the anti-inflammatory efficacy of COD and sabinene by focusing on representative signaling molecules in inflammation-related downstream pathways.

*C. obtusa* leaf essential oil was extracted, and based on its demonstrated sabinene content, its anti-inflammatory effect was investigated (Table 1). First, the toxicity of COD and sabinene was evaluated in RAW 264.7 macrophages (Figure 2a). Subsequently, both compounds were shown to prevent LPS-induced death of RAW 264.7 cells in a dose-dependent manner at a concentration of 200 μg/mL (Figure 2b). The anti-inflammatory potency of COD and sabinene was confirmed based on the ability of both to suppress the expression of iNOS and, thus, NO levels in a dose-dependent manner (Figure 3). COD and sabinene also decreased the expression of IL-1β, IL-6, JNK, and p38 (Figure 4 and Figure 5), as well as that of IL-27, GM-CSF, and IL-1ra (Figure 6) at a concentration of 200 μg/mL.

Our results demonstrated the anti-inflammatory effect of COD, which contains several active substances, including sabinene, through a mechanism involving inhibition of the MAPK axis. Therefore, both COD and sabinene should be further investigated as raw materials for therapeutic applications.

## 4. Materials and Methods

### 4.1. Reagents

Sabinene was purchased from PhytoLab (Vestenbergsgreuth, Germany). Lipopolysaccharide (LPS), Griess reagent, and Triton^®^ X-100 were purchased from Sigma Aldrich (St. Louis, MO, USA). 3-(4,5-dimethylthiazol-2-yl)-2,5-diphenyltetrazolium bromide (MTT) was purchased from Duchefa Biochemie (Haarlem, The Netherlands). Information on the reagents is summarized in Supplementary Table S1.

### 4.2. Antibodies

Anti-iNOS (PA1-036) was purchased from Invitrogen (Carlsbad, CA, USA). Anti-phospho-p44/42 MAPK (Erk1/2) (#9101), anti-p44/42 MAPK (Erk1/2) (#4695), anti-phospho-SAPK/JNK (#9251), anti-SAPK/JNK (#9252), anti-phospho-p38 MAPK (#9211), anti-p38 MAPK (#9212), anti-phospho-NF-κB p65 (#3033), anti-phospho-IκBα (#2859), anti-IκBα (#4814), anti-phospho-JAK1 (#3331), anti-JAK1 (#3332), anti-phospho-STAT1 (#9167), anti-STAT1 (#9172), anti-phospho-STAT3 (#9145), and anti-STAT3 (#30835) were purchased from Cell Signaling Technology (Danvers, MA, USA). Anti-NFκB p65 (sc-8008) and anti-β-actin (sc-47778) were purchased from Santa Cruz Biotechnology (Carlsbad, CA, USA). HRP-conjugated anti-mouse IgG (A21010) and HRP-conjugated anti-rabbit IgG (A21020) were purchased from Abbkine (Wuhan, China). The information on antibodies is summarized in Supplementary Table S2.

### 4.3. Plant Material and Extraction of C. obtusa Essential Oil

*Chamaecyparis obtusa* (Siebold & Zucc.) Endl. (*C. obtusa*) leaves collected from Gwangyang-eup, Gwangyang-si, Jeollanam-do, Korea, were dried naturally and then pulverized with a grinder (Shinil SMX-M41KP, Shinil Electronics, Cheonan-si, Chungcheongnam-do, Republic of Korea). Essential oil was extracted by hydro-distillation using a Clevenger apparatus (Goheung Science, Daegu, Republic of Korea) as follows: 500 mL of distilled water was added to 50 g of *C. obtusa* leaves and heated in a round-bottomed flask at 80 °C. Over a 3-h period, the oil-containing vapor was collected in the condenser, and the condensate was deposited in a Clevenger receiver, where it was separated into oil and hydrolate. Impurities were removed by filtering the oil through a 0.2 μm Millex^®^ syringe filter (Merck, Darmstadt, Germany). The yield of the essential oil was 0.25%.

### 4.4. Gas Chromatography-Mass Spectrometry (GC-MS) Analysis

A total of 100 mg of the sample was added to the dilution solution to a total volume of 10 mL. After homogenization, the sample was diluted 10-fold in dilution solution and injected into a GC-MS system (GC: Agilent 7890A)/MSD: Agilent 5975, Agilent, Santa Clara, CA, USA). Then, 1 μL of sample in dilution solution was injected into an HP-5MS UI column (30 m × 0.25 mm × 0.25 μm, Agilent) at a split ratio of 50:1 (*v*/*v*) and an inlet temperature of 250 °C. Helium served as the carrier gas and was maintained at a flow rate of 1 mL/min. The oven temperature was maintained at 40 °C for 3 min, increased to 200 °C at a rate of 3 °C/min, then to 320 °C at a rate of 15 °C/min, and maintained for 15 min. The interface temperature of the mass spectrometer was 280 °C, and the temperature of the ion source was 250 °C. The mass spectrum had an EI scan range of 35 to 360 *m*/*z* with a scan speed of 1562 μ/s. For materials present in the sample, peaks with an area value of 0.1% or more were integrated based on the largest peak in the TIC, and the mass spectrum was compared with the NIST MS Search 2.0 Mass Spectral Library (National Institute of Standards and Technology, Gaithersburg, MD, USA). Compounds with the highest match value in the NIST library included monoterpenes and sesquiterpenes; the corresponding peaks were classified based on the retention time.

### 4.5. Cell line and Culture

The mouse macrophage cell line RAW 264.7 was obtained from the American Type Culture Collection (ATCC) and grown in Dulbecco’s Modified Eagle’s Medium (DMEM, Capricorn Scientific GmbH, Ebsdorfergrund, Germany) supplemented with 10% fetal bovine serum (FBS, Capricorn Scientific GmbH) and 1% penicillin/streptomycin (Capricorn Scientific GmbH). The cells were incubated in a humidified atmosphere containing 5% CO_2_ at 37 °C (PHCbi CO_2_ Incubator, Tokyo, Japan).

### 4.6. Cell Viability Assay

RAW 264.7 cells (1 × 10^5^ cells/well) were seeded in a 96-well and treated with various concentrations of COD and sabinene for 24 h. Alternatively, they were pretreated for 2 h with COD and sabinene, followed by exposure to 200 ng/mL LPS for 16 h. After treatment, the cells were incubated at 37 °C for 2 h with the MTT reagent. For solubilizing the formazan crystals, DMSO was added to each well. The amount of dissolved formazan crystals was measured at an absorbance of 570 nm using a microplate reader (BioTek Synergy LX, Winooski, VT, USA).

### 4.7. Nitric Oxide (NO) Assay

RAW 264.7 cells (1 × 10^6^ cells/mL) were seeded in a 6-well plate, after being incubated for 24 h, cells were pretreated for 2 h with COD or sabinene at concentrations of 100 and 200 μg/mL, followed by 200 ng/mL LPS for 16 h. To measure NO production, the culture supernatant was mixed with Griess reagent at a ratio of 1:1 (*v*/*v*), and the absorbance was measured at 540 nm using a microplate reader (BioTek Synergy LX).

### 4.8. Immunoblotting

RAW 264.7 cells (1 × 10^6^ cells/mL) were seeded in a 6-well plate and incubated for 24 h. The cells were pretreated for 2 h with COD or sabinene at concentrations of 100 and 200 μg/mL, respectively, followed by stimulation with 200 ng/mL LPS for 4 or 16 h. The cells were washed with cold PBS and lysed in 0.5% Triton X-100 buffer containing protease and phosphatase inhibitors. After incubating the lysate on ice for 10 min, cell debris was removed by centrifugation at 13,000 RPM for 10 min at 4 °C. The supernatant was collected in a new tube, and protein quantification was determined by Bradford assay using Pierce™ Bovine Serum Albumin Standard Ampules (Thermo Fisher, Waltham, MA, USA) and protein assay dye reagent concentrate (Bio-Rad Laboratory, Hercules, CA, USA). Equal amounts of proteins were loaded into 8% sodium dodecyl sulfate-polyacrylamide gel electrophoresis (SDS-PAGE) and transferred to Amersham™ Protran™ 0.45 μm nitrocellulose membranes (Cytiva, Buckinghamshire, UK). The membranes were blocked in Tris-buffered saline with 0.1% Tween 20 (TBST) and 5% skim milk for 1 h at room temperature. Primary antibodies were diluted to 1:1000 (*v*/*v*) in TBST with 1% bovine serum albumin (Bovogen, Keilor East, VIC, Australia) and 0.02% sodium azide, and then incubated overnight at 4 °C. The next day, the membranes were washed with TBST three times and incubated with HRP-conjugated secondary antibodies for 2 h at room temperature. The blots were developed using Clarity™ western ECL substrate (Bio-Rad Laboratory) and visualized using Davinch-Chemi Imager™ CAS-400SM (Davinch-K, Seoul, Republic of Korea).

### 4.9. RNA Isolation and Quantitative Real-Time PCR (RT-qPCR)

After treatment, the cells were washed with cold PBS. To proceed with quantitative real-time PCR analysis, total RNA was isolated using RNAiso Plus (Takara, Shiga, Japan). The total RNA was quantified by purity and concentration measured at the ratio of absorbance at 260/280 nm. The cDNA was synthesized using the ReverTra Ace™ qPCR RT Master Mix kit (Toyobo, Osaka, Japan) according to the manufacturer’s instructions. Quantitative real-time PCR was performed using BlasTaq™ 2X qPCR MasterMix (Applied Biological Materials, Richmond, ON, Canada) according to the manufacturer’s instructions. The amplification reactions using the three-step protocol were detected using a LightCycler^®^ 96 System (Roche, Basel, Switzerland). The fluorescence curves were analyzed using LightCycler^®^ software 1.1, and all expression data were calculated using *β-actin* expression. The primers used in this study were purchased from Bioneer (Daejeon, Republic of Korea), and sequences of primers are listed in Supplementary Table S3.

### 4.10. Cytokine Array

RAW 264.7 cells were pretreated for 2 h with COD or sabinene at a concentration of 200 μg/mL, followed by 200 ng/mL LPS for 4 h. Culture supernatants were collected, and cytokines were measured using the Mouse Cytokine Array Panel kit ARY006 (R&D Systems, Minneapolis, MN, USA).

### 4.11. Dataset Analysis

The dataset used in this study was the GSE206519 dataset. Z-scores were calculated for each gene and visualized by a heat map using Microsoft Excel software 16.67 (Microsoft, Redmond, WA, USA).

### 4.12. Statistical Analysis

Statistical analyses of all data were performed using Microsoft Excel software 16.67. Results are presented as the mean ± standard deviation (SD) of three independent experiments. All significances were determined by an unpaired Student’s *t*-test and considered when the value of *p* < 0.05.

## 5. Conclusions

**Figure 7:**
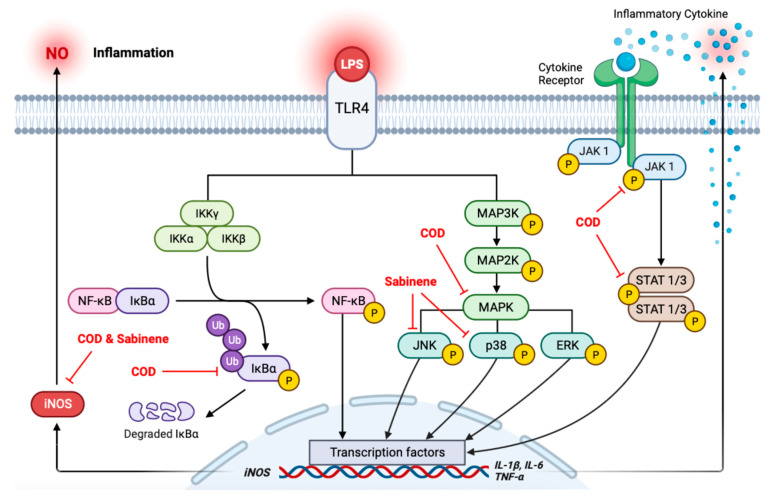
Graphical summary of inflammation in this study. The schematic figure was created using BioRender.com.

In this study, the anti-inflammatory effects of COD and sabinene in LPS-induced RAW 264.7 cells were investigated. Both COD and sabinene reduced the expression of iNOS, phosphorylation of JNK, p38, and expression of pro-inflammatory cytokines IL-1β, IL-6, IL-27, GM-CSF, and IL-1ra, while COD also inhibited the phosphorylation of ERK, IκBα, JAK 1, and STAT 1, 3 (Figure 7).

## Figures and Tables

**Table 1:** Phytocompounds identified in COD by GC-MS.

Chemotype	Compound	R. Time (min)	Relative Abundance
Monoterpene	3-Thujene	10.693	0.53%
	α-Pinene	10.963	1.67%
	Camphene	11.613	0.32%
	Sabinene	12.875	12.34%
	β-Myrcene	13.835	3.62%
	α-Phellandrene	14.371	0.08%
	α-Terpinene	14.973	1.10%
	O-Cymene	15.374	0.87%
	D-Limonene	15.559	6.91%
	γ-Terpinene	17.089	3.96%
	α-Terpinolene	18.543	1.09%
	Trans sabinene hydrate	20.15	0.05%
	Terpinen-4-ol	22.838	2.77%
	α-Terpinenol	23.497	0.35%
	δ 3-Carene	26.658	0.03%
	Bornyl acetate	27.961	10.78%
	Isoterpinolene	28.47	0.07%
	α-Terpyl acetate	30.814	18.78%
Sesquiterpene	β-Elemene	32.642	0.12%
	Trans-α-Bergamotene	33.031	0.08%
	α-Cedrene	33.405	0.06%
	(+)-Epi-Bicyclosesquiphellandrene	33.544	0.20%
	Trans-caryophyllene	33.729	0.44%
	Widdrene	34.218	3.77%
	δ-EIemene	34.434	0.08%
	α-Cubebene	34.905	0.92%
	Humulene	35.144	0.09%
	β-Cubebene	35.579	2.94%
	α-Himachalene	36.131	0.24%
	Germacrene-D	36.331	0.74%
	α-Gurjunene	37.079	2.94%
	Cuparene	37.318	0.26%
	γ-Muurolene	37.673	0.16%
	δ-Cadinene	38.07	1.91%
	α-Muurolene	38.606	0.03%
	Santalene	38.725	6.28%
	Elemol	39.068	0.06%
	γ-Gurjunene	39.338	0.25%
	α-Cedrol	41.023	1.44%
	Cubenol	41.593	0.20%
	8-Epi-γ-eudesmol	41.752	0.17%
	γ-Eudesmol	42.218	2.71%
	τ-Cadinol	42.577	0.31%
	β-Eudesmol	42.899	1.78%
	α-Eudesmol	43.005	2.90%
	Hedycaryol	43.548	0.11%
	β-Elemene	43.914	0.09%
	(+)-α-Bisabolol	44.157	0.11%
	α-Farnesene	45.098	0.05%
Diterpene	Stachen	52.412	2.06%

## Data Availability

The data used and/or analyzed during the current study are available from the corresponding author upon reasonable request.

## Supplementary Materials

The following supporting information can be downloaded at: https://www.mdpi.com/article/10.3390/molecules29051117/s1, Figure S1: Cytokine array blots sorted by exposure time; Table S1: Reagents used in this study; Table S2: Antibodies used in this study; Table S3: Oligonucleotide sequences for the quantitative real-time PCR (RT-qPCR).
