# Supplementary material for: Anti-Inflammatory Effect of Chamaecyparis obtusa (Siebold & Zucc.) Endl. Leaf Essential Oil
Source: Molecules. 2024 Mar 1;29(5):1117. doi: 10.3390/molecules29051117 (PMC10933829; doi:10.3390/molecules29051117)
Supplement: Supplementary file 1 [file molecules-29-01117-s001.zip › molecules-2854484-supplementary.pdf]

Supplementary materials

Anti-Inflammatory Effect of *Chamaecyparis obtusa* (Siebold & Zucc.) Endl. Leaf Essential Oil

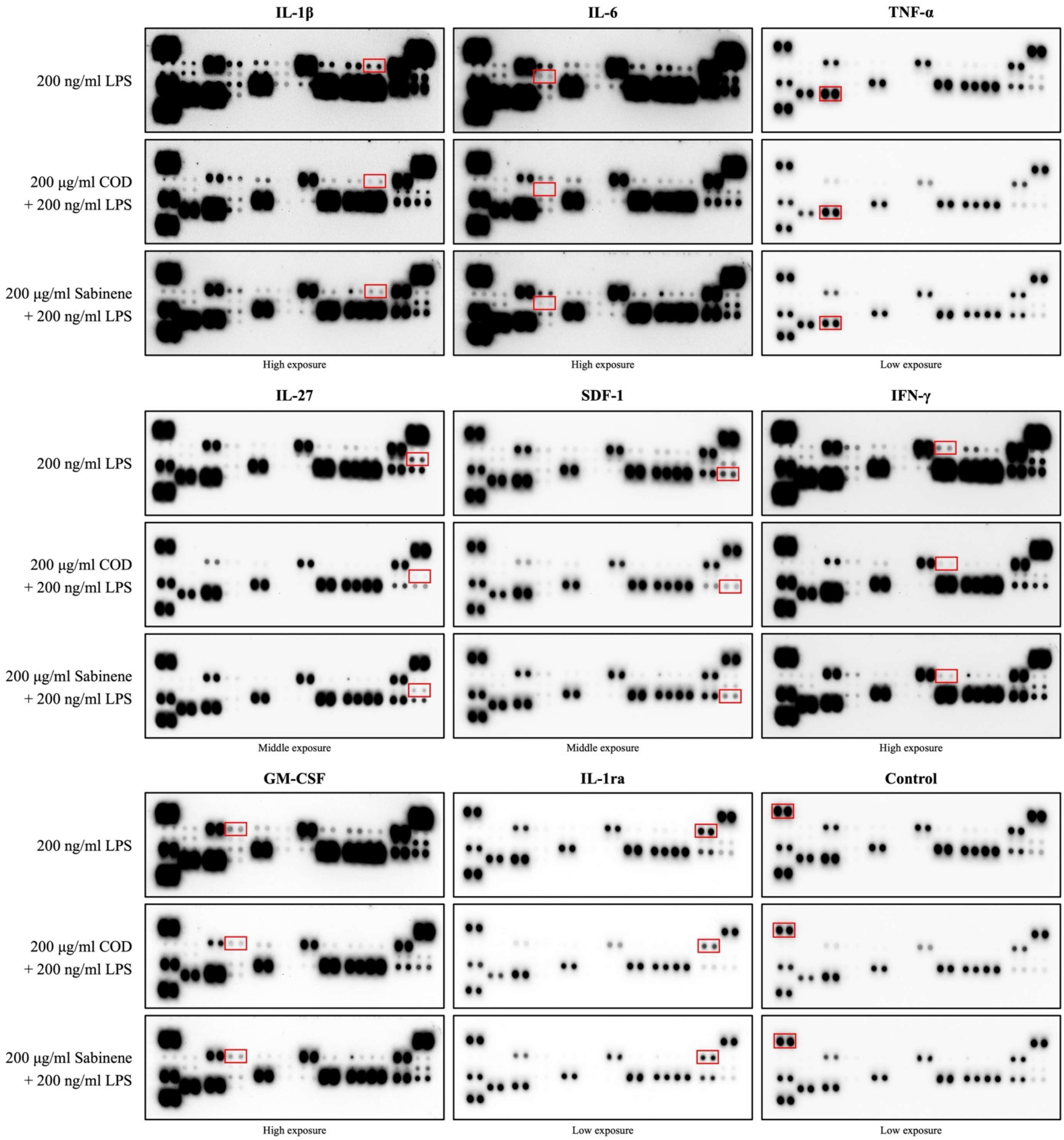

Supplement Figure S1. Cytokine array blots sorted by exposure time. Cytokine array blots were probed with the culture supernatants, untreated or 200 μg/ml COD or 200 μg/ml sabinene. The red squares represent Figure 6b.

Supplement Table S1. Reagents used in this study.

Reagent	Catalog no.	Company
BlasTaq™ 2X qPCR Master Mix	G891	Applied Biological Materials (Richmond, Canada)
Skim Milk	232100	BD Difco (Sparks, MD, USA)
Nutrient Agar (NA)	213000	
Nutrient Broth (NB)	234000	
Tryptic Soy Agar (TSA)	236950	
Tryptic Soy Broth (TSB)	211825	
Clarity™ Western ECL Substrate	#1705060	Bio-Rad Laboratory (Hercules, CA, USA)
Protein Assay Dye Reagent Concentrate	#5000006	
Bovine Serum Albumin	BSAS 0.1	Bovogen (Keilor East, VIC, Australia)
Dulbecco's Modified Eagle's Medium (DM EM)	DMEM-HPA	Capricorn Scientific GmbH (Ebsdorfergrund, Germany)
Fetal Bovine Serum (FBS)	FBS-22A	
Penicillin/Streptomycin (100X)	PS-B	
Amersham™ Protran™ 0.45 NC Membrane	10600002	Cytiva Amersham (Buckinghamshire, UK)
3-(4,5-Dimethylthiazol-2-yl)-2,5-Diphenylte trazolium Bromide (MTT)	M1415	Duchefa Biochemie (Haarlem, Netherlands)
Sabinene	PHL82342	PhytoLab (Vestenbergsgreuth, Germany)
Mouse Cytokine Array Panel kit	ARY006	R&D Systems (Minneapolis, MN, USA)
Griess reagent	G4410	Sigma Aldrich (St.Louis, MO, USA)
Lipopolysaccharide (LPS)	L3012	
Triton® X-100	9002-93-1	
RNAiso Plus	9109	Takara (Shiga, Japan)
ReverTra Ace™ qPCR RT Master Mix with gDNA Remover	FSQ-301	Toyobo (Osaka, Japan)

Supplement Table S2. Antibodies used in this study.

Antibody	Catalog no.	Company
Phospho-p44/42 MAPK (Erk1/2)	#9101	Cell Signaling Technology (Danvers, MA, USA)
p44/42 MAPK (Erk1/2)	#4695	
Phospho-SAPK/JNK	#9251	
SAPK/JNK	#9252	
Phospho-p38 MAPK	#9211	
p38 MAPK	#9212	
Phospho-NF-κB p65	#3033	
Phospho-IκBα	#2859	
IκBα	#4814	
Phospho-JAK1	#3331	
JAK1	#3332	
Phospho-STAT1	#9167	
STAT1	#9172	
Phospho-STAT3	#9145	
STAT3	#30835	
iNOS	PA1-036	Invitrogen (Carlsbad, CA, USA)
NFκB p65	sc-8008	Santa Cruz Biotechnology (Carlsbad, CA, USA)
β-actin	sc-47778	
HRP, Goat Anti-Mouse IgG	A21010	Abbkine (Wuhan, China)
HRP, Goat Anti-Rabbit IgG	A21020	

Supplement Table S3. Oligonucleotide sequences for the quantitative real-time PCR (RT-qPCR).

Gene name		Sequence (5' → 3')
iNOS (mouse)	Forward	CAGCACAGGAAATGTTTCAGC
	Reverse	TAGCCAGCGTACCGGATGA
TNF-α (mouse)	Forward	TATGGCTCAGGGTCCAACTC
	Reverse	CTCCCTTTGCAGAACTCAGG
IL-1β (mouse)	Forward	TTGACGGACCCCAAAAGATG
	Reverse	AGAAGGTGCTCATGTCCTCA
IL-6 (mouse)	Forward	GGTGACAACCACGGCCTTCCC
	Reverse	AAGCCTCCGACTTGTGAAGTGGT
β-actin (mouse)	Forward	TGTCCACCTTCCAGCAGATGT
	Reverse	AGCTCAGTAACAGTCCGCCTAGA
